# Bowel function after laparoscopic right hemicolectomy: a randomized controlled trial comparing intracorporeal anastomosis and extracorporeal anastomosis

**DOI:** 10.1007/s00464-021-08854-8

**Published:** 2021-11-03

**Authors:** Piotr Małczak, Michał Wysocki, Magdalena Pisarska-Adamczyk, Piotr Major, Michał Pędziwiatr

**Affiliations:** 1 Department of Medical Education, Jagiellonian University Medical College, Kraków, Poland; 2 2Nd Department of General Surgery, Jagiellonian University Medical College, Kopernika 21, 31-501 Kraków, Poland

**Keywords:** Laparoscopic right hemicolectomy, Bowel function, Anastomosis

## Abstract

**Background:**

The laparoscopic right hemicolectomy is the standard surgical treatment for right-sided colon cancer. The continuity of the digestive tract is restored through ileocolic anastomosis which can be performed extracorporeally or intracorporeally. The study aimed to compare both anastomotic techniques in laparoscopic right hemicolectomy.

**Materials and methods:**

A single-blinded two-armed randomized control trial with 1:1 parallel allocation carried out from 2016 to 2020 in a single center. The follow-up period was 30 days. Compared interventions involved extracorporeal and intracorporeal ileocolic anastomosis in laparoscopic right hemicolectomy. The main outcome of the study was bowel recovery measured as the time to the first stool. Other outcomes involved the time to the first flatus, morbidity, and duration of surgery.

**Results:**

One hundred and seventeen patients undergoing a laparoscopic right hemicolectomy with curative intent were eligible for the trial. Eight patients refused to participate. One hundred and two patients were analyzed, 52 in the intracorporeal group and 50 in the extracorporeal group. The groups did not differ in terms of cancer stage or body mass index, but did differ in age and sex. Intracorporeal anastomosis was associated with a shorter time to the first stool than extracorporeal, 32.8 h (26.0–43.7) vs. 41.7 (35.9–50.0), *p* = 0.017. There was no significant difference in the time to the first flatus, 30 h (23.2–42.3) vs. 26.6 h (21.8–37.3), *p* = 0.165. Similarly, overall complications did not differ (EC 12/50 vs. IC 10/52, *p* = 0.56). There were no differences in length of surgery, 190 min (150–230) and 190 min (180–220), *p* = 0.55.

**Conclusion:**

Intracorporeal ileocolic anastomosis following laparoscopic right hemicolectomy results in slightly faster bowel recovery, with no differences in morbidity and duration of surgery.

Since its introduction to colorectal surgery, laparoscopy has been proven to improve many short- and long-term results [1–4]. Laparoscopic right hemicolectomy is a well-established procedure for the treatment of right-sided colon neoplasms [5, 6]. Following excision of the bowel, an anastomosis between the small and large intestine needs to be created. Extracorporeal anastomosis (EC) is the gold standard for restoring continuity; however, intracorporeal (IC) anastomosis is also a feasible technique that has been proven to be equally safe and efficient in numerous observational studies. Some of these show the superiority of IC, while others show the opposite [7–9]. While the potential feasibility and benefits of IC have been shown in previous studies, randomized control trials (RCT) confirming which method produces better results are still lacking [10, 11]. This RCT aims to compare short-term (30 days) clinical outcomes between IC and EC in totally laparoscopic right hemicolectomy.

## Material and methods

### Design

This is a single-center, single-blinded, randomized study comparing outcomes in patients undergoing elective surgery in a tertiary referral unit from 2016 to 2020. The study is reported in line with Consolidated Standards of Reporting Trials (CONSORT) Guidelines [12]. It was designed as a parallel-group trial with a 1:1 allocation ratio. All surgeons were high volume laparoscopic colorectal surgeons (over 150 laparoscopic colorectal cancer resections) skilled in performing both IC and EC procedures. All consecutive patients aged 18 years or older with a benign or malignant right-sided colon neoplasm that were undergoing a laparoscopic right hemicolectomy were considered. Exclusion criteria were stage IV disease, conversion, inflammatory bowel diseases, corticosteroid intake, immunodeficiency, emergency surgery, neoadjuvant therapy, a lack of technical possibility to perform both types of anastomosis, and larger procedures, such as extended right colectomies. All procedures followed the ethical standards of the responsible committee on human experimentation (institutional and national) and the Sixth Revision (Fortaleza) of the 1975 Declaration of Helsinki. The study was approved by an independent ethics committee of the Jagiellonian University, Krakow, Poland no. 122.6120.82.2017. The study protocol was registered at www.ClinicalTrials.gov (NCT04578405).

The primary outcome was the time from the end of surgery to the first stool. Secondary outcomes were the time to the first flatus, the duration of the surgery, the length of the hospital stay (LOS), postoperative morbidity, and readmissions. Patients were randomized and allocated using sealed envelopes after signed consent was obtained. Patients were blinded to the selected intervention. The data were collected by staff that were not involved in the patient’s management during the RCT. The statistician who analyzed the data was blinded as well.

### Surgical technique

Following anesthesia, patients were placed in a low lithotomy position. After creating pneumoperitoneum (12 mmHg), five trocars (5–12 mm) were inserted. A standard 10-mm 30° laparoscope camera was used. The procedure consisted of medial to lateral laparoscopic mobilization of the right colon followed by dissection of the bowel together with blood vessels and the accompanying mesocolon and lymph nodes. For vessel sealing we used either Ethicon Harmonic ACE®, Olympus Thunderbeat®, or Medtronic LigaSure® (according to the surgeon’s preference). Greater vessels were clipped with titanium clips. In cases of EC, the specimen was extracted via transverse mini-laparotomy in epigastrium, which was protected with Applied Medical Alexis Wound Protector® and used for the extracorporeal anastomosis. In cases of IC, the specimen was extracted via Pfannenstiel incision. EC anastomosis was hand-sewn with a single-layer continuous suture using 4-0 PDS. IC anastomosis was performed using a 60 mm stapler and the hole was closed using a V-loc suture. Neither nasogastric tubes nor drains were used routinely. Finally, bilateral *transversus abdominis plane* block (TAP block) under ultrasound guidance was used at the end of the surgery (20 ml of 0.25% bupivacaine solution on each side).

Perioperative care protocol based on ERAS protocol was used in all patients. Detailed information on the protocol in our department was described in previous studies [13, 14]. There was no mechanical preparation of the bowel. Preoperative antibiotic involved neomycin and metronidazole orally the day before surgery and cefuroxime with metronidazole intravenously 30 min before surgery patient received meglumine amidotrizoate and sodium amidotrizoate (Gastrografin®) twice a day orally as a propulsive agent after surgery. Early oral feeding (oral nutritional supplement in the evening on the day of surgery, light hospital diet and oral nutritional supplements on the first postoperative day, full hospital diet in the second postoperative day). Patients were discharged from the hospital when they were fully mobilized, tolerated oral diet, did not require any intravenous medication, and did not require help of other people.

### Statistics

The minimum number of participants per arm was calculated for the primary outcome assuming power of 80% and alpha 0.05. The time to the first stool for EC was based on a study by Milone et al. and our clinical data [9]. The sample size was 50 patients per arm in order to show a difference of at least 20%, with an additional 15% surplus. Statistical analysis was performed using StatSoft Statistica version 13.0 software. Normal distribution of continuous variables was tested with the *χ*^2^ test. Variables with non-normal distribution were compared using the Mann–Whitney *U* test. Categorical variables were compared with a *χ*^2^ test. Results were considered statistically significant when the *p* value was < 0.05.

## Results

**Fig. 1:**
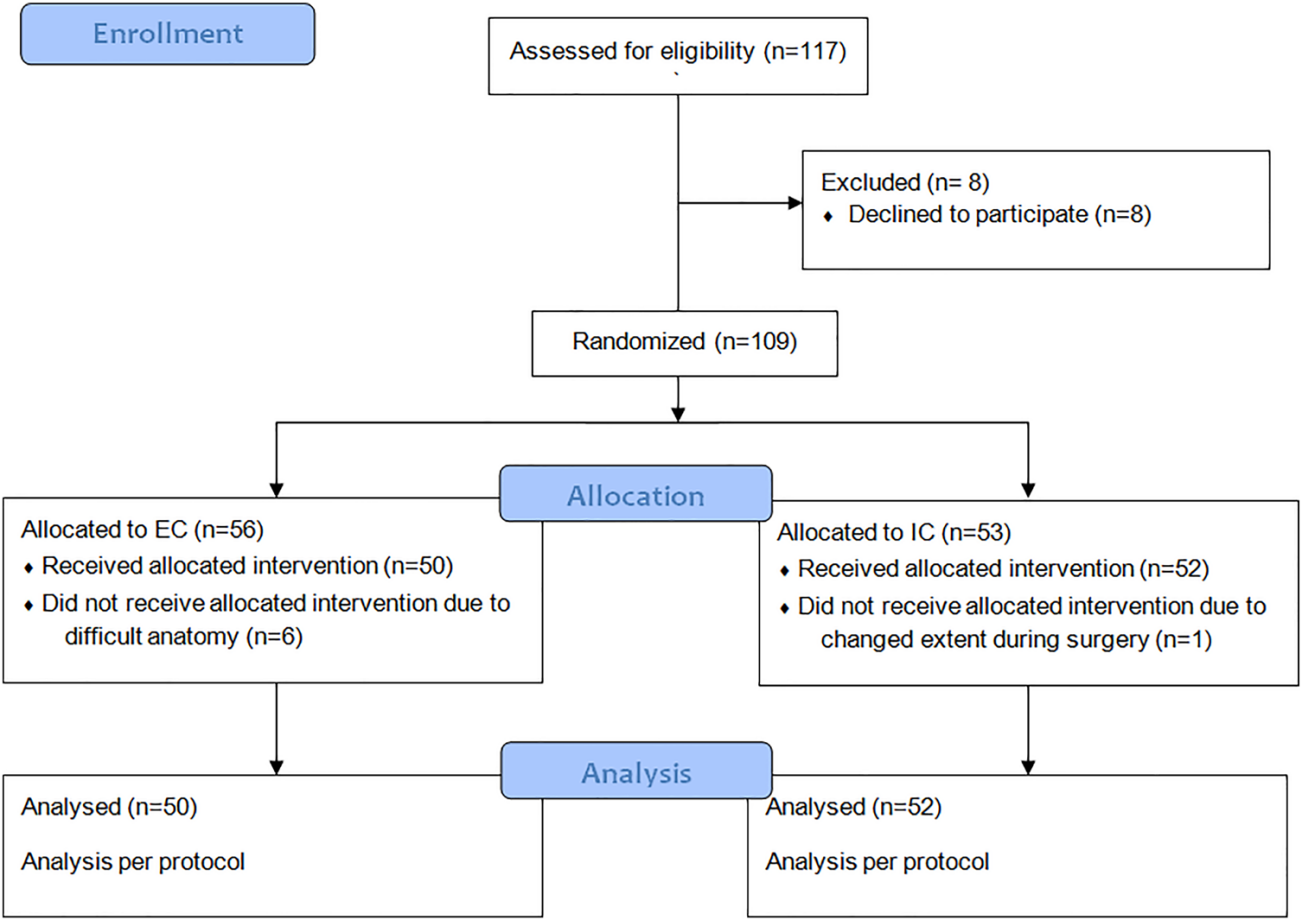
Patient flow through the study

In the analyzed period of time, 117 patients undergoing laparoscopic right hemicolectomy were potentially eligible for inclusion. Of these, eight did not agree to participate in the study. The remaining 109 patients were then randomized, 56 to EC and 53 to IC. One patient in IC was excluded from analysis due to a subtotal colectomy (intraoperative decision) and six patients in EC were excluded due to difficulty in performing both types of anastomosis because of a shortened mesentery and a thick abdominal wall (instead IC anastomosis has been done). All excluded patients were male. This resulted in a total of 102 patients in the final analysis, 52 patients in IC and 50 in EC, as shown in Fig. 1. Table 1 presents baseline characteristics of the analyzed group.

**Table 1:** Comparison of patient-related data

	External	Internal	*p*-value
*n* (%)	50 (49%)	52 (51%)	n/a
Males/females, *n* (%)	28/22 (56%/44%)	16/36 (31%/69%)	**0.01**
Median age, years (IQR)	69 (60–72)	74 (65–80)	**0.01**
Median BMI, kg/m^2^ (IQR)	27.6 (24.4–31.2)	28.4 (23.5–32.5)	0.41
Localization of cancer, *n* (%)			
Caecum	30 (60%)	14 (27%)	**0.009**
Ascending colon	14 (28%)	22 (42%)	
Hepatic flexure	4 (8%)	12 (23%)	
Transverse colon	2 (4%)	2 (4%)	
Cecum + hepatic flexure	0	2 (4%)	n/a

*n/a* not applicable

Patients in the IC group had a significantly shorter time to first stool, 32.8 h (26.0–43.7) vs. 41.7 (35.9–50.0), *p* = 0.017. There was no significant difference in the time to the first flatus (30 h vs. 26.6 h), *p* = 0.165. Similarly, the groups did not vary in terms of length of operation (190 min vs. 190 min, *p* = 0.55), blood loss (EC 90 ml vs. IC 50 ml, *p* = 0.4), overall complications (EC 12/56, 24% vs. IC 10/52, 19%, *p* = 0.56), or minor and major complications (*p* = 0.99 and *p* = 0.37, respectively). Four patients in EC group had to be re-operated on, two due to anastomotic leakage and two due to bleeding. There were no reoperations following IC. Two IC patients were admitted to the ICU for observation due to their general health condition prior to surgery. There were no cases of mortality. All resections were oncologically R0. There were no differences in tumor grades and stages between the groups. In the majority of the patients, the stage was T3. Detailed information is presented in Table 2.

**Table 2:** Comparison of surgical and clinical outcomes

	External	Internal	*p*-value
Median time interval between operation and passing stool, hours (IQR)	41.7 (35.9–50)	32.8 (26–43.7)	**0.02**
Median time interval between operation and passing gas, hours (IQR)	30 (23.2–42.3)	26.6 (21.8–37.3)	0.17
Median LOS, days (IQR)	4 (3–5)	4 (3–6)	0.56
Median operative time, min (IQR)	190 (150–230)	190 (180–220)	0.55
Median blood loss, ml (IQR)	90 (50–100)	50 (45–150)	0.4
Median length of mini-laparotomy, cm (IQR)	7.2 (7–8)	6.15 (6–6.5)	** < 0.001**
Conversion, *n* (%)	0	0	n/a
Perioperative mortality, *n* (%)	0	0	n/a
Perioperative morbidity, *n* (%)	12 (24%)	10 (19%)	0.56
CD 1–2, *n* (%)	10 (20%)	10 (19%)	0.99
Pulmonary complications	4 (8%)	8 (15%)	n/a
Ileus	5 (10%	2 (3.85%)	n/a
Hematoma	1 (2%)	0	n/a
CD 3–5, *n* (%)	4 (8%)	2 (4%)	0.37
Bleeding, *n* (%)	2 (4%)	0	n/a
Readmissions, *n* (%)	4 (8%)	0	n/a
Anastomotic leakage, *n* (%)	2 (4%)	0	n/a
Reoperations, *n* (%)	4 (8%)	0	n/a
R0 resections, *n* (%)	50 (100%)	52 (100%)	n/a

*n/a* not applicable

## Discussion

Intracorporeal and extracorporeal anastomoses are both well-established techniques for restoring digestive tract continuity in laparoscopic right hemicolectomy. There are many observational studies showing the benefits of both, while there are very few RCTs. This prompted us to perform an RCT comparing IA with EA. The optimal question for an RCT would confirm which technique has a lower anastomotic leakage rate; however, the low rate of this adverse event would require an extremely high number of participants, which makes it difficult to conduct. Since laparoscopy has reduced the stress caused by the surgery reducing morbidity and length of hospital stay, recovery of the bowel functions is one of the main factors in determining early discharge. In our observations prior to this study, IC was associated with faster bowel recovery. This may be due to several factors, including reduced bowel manipulation and mesentery traction. To minimize additional factors influencing the outcome, we excluded patients with history of inflammatory bowel diseases, corticosteroid intake, immunodeficiency, or neoadjuvant therapy.

Our study showed that IC has a significantly shorter time for bowel recovery in comparison to EC, 33 h vs. 42 h. Allaix et al. showed similar results in favor of IC in their study, 4 vs. 4.5 days. Bollo et al. showed even shorter times in favor of IC, 2.3 vs. 3.3 days. Our study did not show significant differences in the time to the first flatus, while Allaix showed a shorter time for IC. The majority of studies showed a shorter length of incision in IC technique.

In a recent large observational study by Anania et al. [15], it was seen that IC is associated with a longer operative time. Conversely, a meta-analysis by Selvy et al. [16] showed no differences in the duration of the surgery. We did not find significant differences in the length of the operations. It is well known that totally laparoscopic surgeries are more technically demanding and that the learning curve is steeper than it is for open procedures, thus the surgeries in the analysis should be performed by specialists who have performed the necessary number of surgeries [17]. We only analyzed patients in which both techniques (intra and extracorporeal anastomosis) were possible.

Length of hospital stay in our patients did not differ between the groups. Both available RCTs by Allaix and Bollo did not find differences either. In a recent large multi-center observational study by Saleh et al. [18], authors showed results in favor of IC in terms of LOS. Similar results were shown by Selvy et al. [16] in a meta-analysis. In our opinion, these differences between studies may be caused by ERAS protocol. In units where ERAS protocol has been introduced and maintained at high compliance the LOS is generally shorter [19, 20].

There were no differences in terms of major (CD 3–5) and minor complications (CD 1–2). Similar results were presented by Allaix et al., whereas Bollo et al. showed higher rate of minor complications in the EC group. Most recent meta-analysis showed no significant differences in morbidity.

Laparoscopic right hemicolectomy has evolved through the addition of complete mesocolic excision (CME). The technical difficulties regarding proper dissection of vessels will undoubtedly prolong the surgery; however, the oncological benefit is unequivocal [21]. Still, the anastomotic part of the procedure remains the same; thus, in our opinion, experiences from previous studies and this RCT can be extrapolated to apply to a CME hemicolectomy.

The limitations of this study are typical of single-center studies. Additionally, bowel functioning was only assessed by time to stool and flatus, and time of normalization of oral intake was not analyzed. Patients' obstipation history was not analyzed in this study. Another limiting factor is our perioperative patient care protocol which may influence the postoperative course to some extent.

## Conclusion

Intracorporeal anastomosis following a laparoscopic right hemicolectomy leads to faster recovery of bowel functions, as compared to extracorporeal anastomosis, without prolonging the surgery or increasing morbidity.
